# ACE2 and TMPRSS2 expression in patients before, during, and after SARS-CoV-2 infection

**DOI:** 10.3389/fcimb.2024.1355809

**Published:** 2024-03-28

**Authors:** Henrique Borges da Silva Grisard, Marcos André Schörner, Fernando Hartmann Barazzetti, Julia Kinetz Wachter, Manoela Valmorbida, Glauber Wagner, Gislaine Fongaro, Maria Luiza Bazzo

**Affiliations:** ^1^ Laboratório de Biologia Molecular, Microbiologia e Sorologia, Departamento de Análises Clínicas, Centro de Ciências da Saúde, Universidade Federal de Santa Catarina, Florianópolis, Brazil; ^2^ Laboratório de Bioinformática, Departamento de Microbiologia, Imunologia e Parasitologia, Centro de Ciências Biológicas, Universidade Federal de Santa Catarina, Florianópolis, Brazil; ^3^ Programa de Pós-Graduação em Biotecnologia e Biociências, Departamento de Microbiologia, Imunologia e Parasitologia, Universidade Federal de Santa Catarina, Florianópolis, Brazil; ^4^ Programa de Pós-Graduação em Farmácia, Departamento de Análises Clínicas, Universidade Federal de Santa Catarina, Florianópolis, Brazil; ^5^ Laboratório de Virologia Aplicada, Departamento de Microbiologia, Imunologia e Parasitologia, Centro de Ciências Biológicas, Universidade Federal de Santa Catarina, Florianópolis, Brazil

**Keywords:** SARS-CoV-2, host-parasite, pathogenicity, ACE2, TMPRSS2

## Abstract

During the SARS-CoV-2 pandemic angiotensin-converting enzyme 2 (ACE2) and transmembrane serine protease 2 (TMPRSS2) were constantly under the scientific spotlight, but most studies evaluated ACE2 and TMPRSS2 expression levels in patients infected by SARS-CoV-2. Thus, this study aimed to evaluate the expression levels of both proteins before, during, and after-infection. For that, nasopharyngeal samples from 26 patients were used to measure ACE2/TMPRSS2 ex-pression via qPCR. Symptomatic patients presented lower ACE2 expression levels before and after the infection than those in asymptomatic patients; however, these levels increased during SARS-CoV-2 infection. In addition, symptomatic patients presented higher expression levels of TMPRSS2 pre-infection, which decreased in the following periods. In summary, ACE2 and TMPRSS2 expression levels are potential risk factors for the development of symptomatic COVID-19, and the presence of SARS-CoV-2 potentially modulates those levels.

## 1 Introduction

Viruses belonging to the Coronaviridae family have caused several infections, such as the Middle East Respiratory Syndrome (MERS) caused by MERS Coronavirus, which emerged in the Arabian Peninsula around the year 2012, and Severe Acute Respiratory Syndrome (SARS) caused by SARS Coronavirus (SARS-CoV), both of which are highly infectious and potentially pathogenic. During the last 4 years a new coronavirus, SARS-CoV-2, has been causing an infection named coronavirus disease 2019 (COVID-19), which is transmitted via the upper airways and causes various symptoms, mainly affecting the respiratory, digestive, and neurological systems (Wu et al., 2020; Gasmi et al., 2021).

SARS-CoV-2 is an enveloped virus with an RNA (+) genome, whose process of cellular infection occurs via binding of the viral spike protein (S) to the host receptor, angiotensin-converting enzyme 2 (ACE2), and its cleavage by transmembrane serine protease 2 (TMPRSS2) into the S1 and S2 subunits, allowing viral entry into the host cell (Benetti et al., 2020; Lieberman et al., 2020). ACE2 is a zinc metallopeptidase (Ferrario et al., 2005) widely expressed in human tissues (Ni et al., 2020; Beyerstedt et al., 2021), particularly in the vascular, pulmonary, and intestinal epithelia (Hamming et al., 2004). ACE2 belongs to the renin–angiotensin–aldosterone system (RAAS) which is essential for maintaining blood vessel tonicity, arterial pressure, and sodium/potassium balance (Muñoz-Durango et al., 2016; Patel et al., 2017). The RAAS produces its effects via the effector molecule, angiotensin II (ang II), which is a potent vasoconstrictor when not con-verted by ACE2, but by its AT1 receptor. However, to balance this vasoconstriction effect caused by ang II, the same protein can be converted by ACE2 into angiotensin 1-7 (ang-(1-7)), another effector molecule which is a vasodilator with effects contrary to those of ang II (Deng et al., 2001; Turner et al., 2004; Rüster and Wolf, 2006; Benigni et al., 2010; Kuba et al., 2013).

Although the physiological role of TMPRSS2 is not well-described (Antalis et al., 2011), its expression levels are modulated by androgenic pathways (Baughn et al., 2020), and its importance is well-recognized in the viral cycle of SARS-CoV-2 and SARS-CoV. Both, ACE2 and TMPRSS2, have gained considerable importance and interest on their relevance in the pathogenicity of COVID-19, but these studies utilized samples from patients who were already infected with SARS-CoV-2 and did not have a reference sample from the same patient at another time. Therefore, this study aimed to evaluate the expression levels of ACE2 and TMPRSS2 in nasopharyngeal samples before, during, and after SARS-CoV-2 infection, to understand the influence of these levels on the pathogenicity of COVID-19.

## 2 Materials and methods

### 2.1 Ethics approval and consent to participate

The Institutional Ethics Review Board of the Federal University of Santa Catarina (UFSC) approved this study under the reference number CAAE:57722022.2.0000.0121. Before sampling, the included patients signed the consent form, which was also approved by the same review board.

### 2.2 Clinical samples

We were able to obtain samples of the same patients in different periods, so in total, 65 nasopharyngeal samples were obtained from 26 patients (8 asymptomatic and 18 symptomatic) between April 2020 and January 2022. Written informed consent was provided by the patients and clinical data were provided at the time of sampling. We considered patients with at least one of the following symptoms as symptomatic: fever, coughing, nausea, cephalgia, nasal congestion, diarrhea, arthralgia, myalgia, loss of smell/taste, sinusitis, and night sweats. Each patient enrolled in this study had at least two samples from two different periods (**Table 1**), allowing the comparison of expression between different periods. The lack on data of RAAS blocker usage is a limitation of this study, given that only 9 patients declared to have not used RAAS blockers, whilst for the other 18 we weren’t able to obtain data.

**Table 1:** Description of the number of patients in the four categories of sampling periods and the average timespan between sampling periods.

Sampling periods	Patients	Symptomatic patients	Asymptomatic patients	Average time between samplings
Pre and COVID-19	11	8	3	14 weeks
COVID-19 and Post	13	10	3	22 weeks
Pre and Post	14	8	6	33 weeks
Pre, COVID-19, and Post	8	6	2	10 weeks (Pre and COVID-19)/28 weeks (COVID-19 and Post)/38 weeks (Pre and Post)

### 2.3 Total RNA extraction and reverse transcription

Total RNA from nasopharyngeal samples was isolated using the RNeasy Mini Kit (QIAGEN^®^, USA) following the manufacturer’s protocol, except for the initial centrifugation, which was performed at 1300 × g for 40 min. Following purification, the total RNA was quantified using Nanovue Plus (General Electric^®^, USA). The known RNA concentrations were then used to dilute the samples to the same concentration using RNase-free water, equalizing the RNA input for the following steps: the quantified and diluted RNA was subjected to reverse transcription to produce complementary DNA (cDNA), for which a high-capacity cDNA Reverse Transcription Kit (Applied Biosystems^®^, USA) was used, following the manufacturer’s protocol.

### 2.4 Absolute quantification of *ACE2* and *TMPRSS2* genes

For the absolute quantification of ACE2 and TMPRSS2 expression levels, real-time polymerase chain reaction (qPCR) was used to amplify both genes encoding ACE2 and TMPRSS2 alongside the beta-2-microglobulin (B2M) gene, a well-conserved human gene used as an amplification control. qPCR was performed using GoTaq ™ Probe Master Mix (Promega™, USA) with the following probe assays: PrimePCR™ Probe Assay: ACE2, Human (BioRad™, USA); TMPRSS2, Human (BioRad™, USA); and B2M, Human (Bio-Rad™, USA) (amplicon sequences listed in **Table 2**). Plasmids with known concentrations were designed and amplified along with clinical samples to provide a standard curve for absolute quantification. For each plasmid, the probe amplicon sequence (**Table 2**) was submitted to the Basic Local Alignment Search Tool (BLAST) - NCBI to obtain the reference gene from which the sequence was obtained. Using the reference genes (NCBI: ACE2 - NM_021804.3/TMPRSS2 - NM_001135099.1/B2M - NM_004048.4) 20 base pairs (bp) were added to the 5′ and 3′ ends of the probe’s amplicon sequence, to ensure primer annealing. An amplicon sequence with 20 bp at each end resulted in a plasmid target sequence (**Table 2**).

**Table 2:** Specifications of the probe’s amplicon nucleotide sequences and designed plasmids amplicon nucleotide sequences used in this study.

Amplicon	Sequence 5′- 3′
*PrimePCR™ Probe Assay: ACE2, Human (BioRad^®^)*	TAATGCATGCCATTCTCAATCCTTGCAGC TACACCAGTTCCCAGGCACTGTCCTTACA AGTGATCCATCCATATTCCATACAAGATC CAACACTTGCTCCAATGATATATCC
*PrimePCR™ Probe Assay: TMPRSS2, Human (BioRad^®^)*	CAGACAAGTTCACTGTTTAATAAAAATG AAGTGACCTCTGAATCATCTCTAAGAGT AAATCATGCACGGGGAAGCAAAACCAG CCCCATTGTTTTCTTGTAAAACGACGTC AAGGACGAAGACCATGTGGATTAGCCG TCTGCCCTCATTTGTCGATAAATCCAGT CCGTGAATACCATC
*PrimePCR™ Probe Assay: B2M, Human (BioRad^®^)*	TCCGTGGCCTTAGCTGTGCTCGCGCTAC TCTCTCTTTCTGGCCTGGAGGCTATCCA GCGTACTCCAAAGATTCAGGTTTACTCA CGTCATCCAGCAGAGAATGGAAAGTCA AATTTCCTGAATTGCTATGTGTCTGGGT TTCATCCATCCGAC
*ACE2* plasmid	ATGGATTAAATGAAAGTGAGCTAATGCA TGCCATTCTCAATCCTTGCAGCTACACC AGTTCCCAGGCACTGTCCTTACAAGTGA TCCATCCATATTCCATACAAGATCCAAC ACTTGCTCCAATGATATATCCTTGTTTTC AACTTCAGAAAT
*TMPRSS2* plasmid	ATGGCAGAGAGTGCCAAAGCCAGACAA GTTCACTGTTTAATAAAAATGAAGTGAC CTCTGAATCATCTCTAAGAGTAAATCAT GCACGGGGAAGCAAAACCAGCCCCATT GTTTTCTTGTAAAACGACGTCAAGGACG AAGACCATGTGGATTAGCCGTCTGCCCT CATTTGTCGATAAATCCAGTCCGTGAAT ACCATCACATTCCCGTACACTCCTGG
*B2M* plasmid	TTCGGGCCGAGATGTCTCGCTCCGTGGC CTTAGCTGTGCTCGCGCTACTCTCTCTTT CTGGCCTGGAGGCTATCCAGCGTACTCC AAAGATTCAGGTTTACTCACGTCATCCA GCAGAGAATGGAAAGTCAAATTTCCTGA ATTGCTATGTGTCTGGGTTTCATCCATCC GACATTGAAGTTGACTTACTGAA

ACE2, angiotensin-converting enzyme 2; TMPRSS2, transmembrane serine protease 2; B2M, beta-2-microglobulin.

### 2.5 Statistical analyses

Samples and plasmids were amplified in triplicate, and those with a standard deviation ≥ 0.5 for the threshold cycle values were not considered for the analysis. A Fisher-Snedecor F Distribution was applied using GraphPad Prism^®^ to compare the expression levels between groups with varying amounts of samples.

## 3 Results

### 3.1 *ACE2* and *TMPRSS2* expression levels

**Figure 1:**
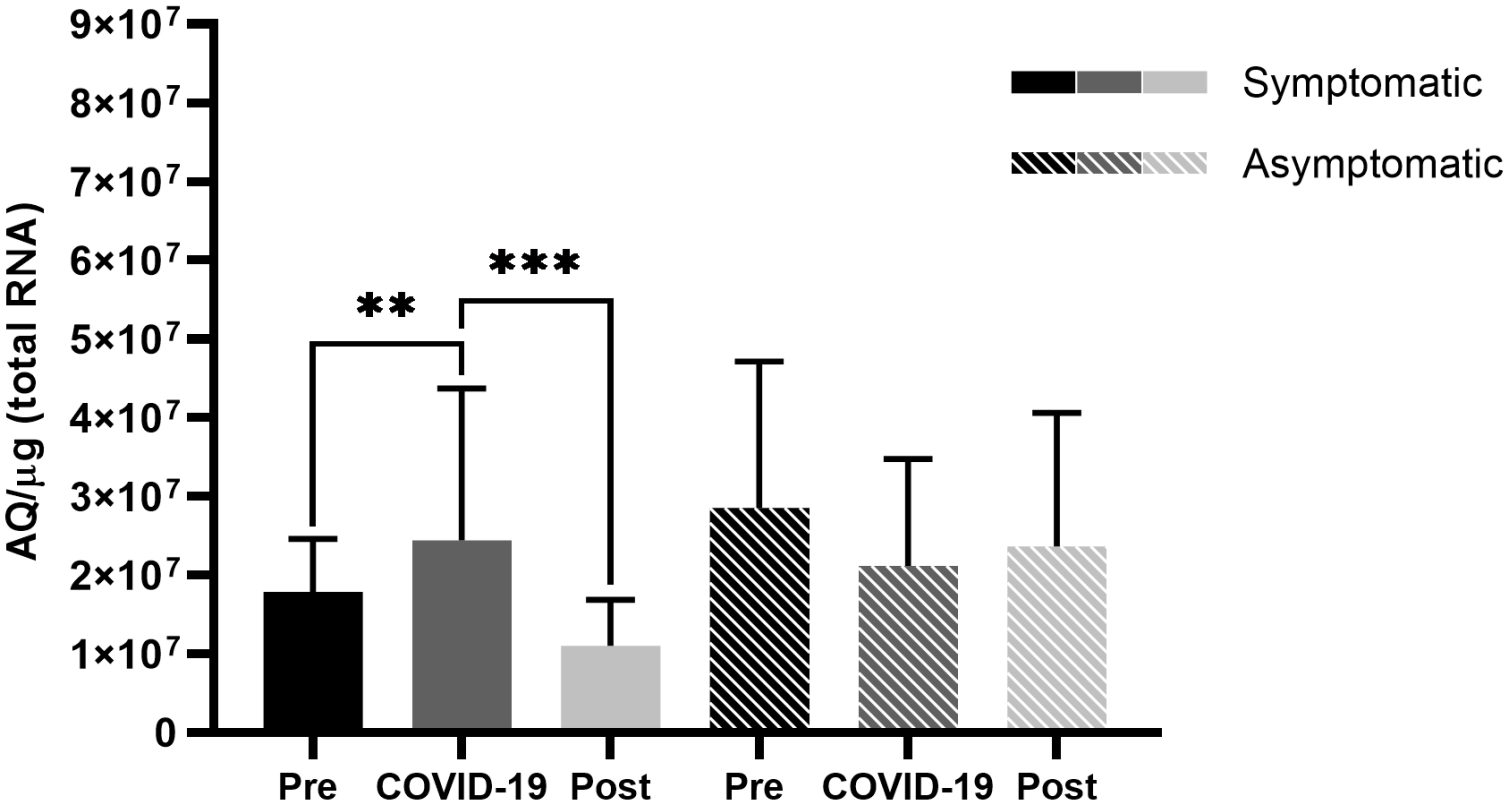
Angiotensin-converting enzyme 2 (ACE2) expression in the nasopharyngeal samples of symptomatic and asymptomatic patients pre-, during-, and post- infection with severe acute respiratory syndrome coronavirus 2 (SARS-CoV-2). Comparison of ACE2 expression between symptomatic and asymptomatic patients at different sampling points. Bars represent Standard Error of the Mean (SEM), and Y axis plots Absolute Quantity per microgram of total RNA (AQ/µg). Using the Fisher-Snedecor F distribution, significant differences were established within the groups, represented by **p < 0.01, ***p < 0.001.

Analysis of ACE2 expression behavior among symptomatic patients (**Figure 1**) revealed low expression levels of before infection, which increased significantly during SARS-CoV-2 infection (*p* < 0.01) and decreased significantly after the infection (*p* < 0.001), returning to approximately the levels observed before the infection.

**Figure 2:**
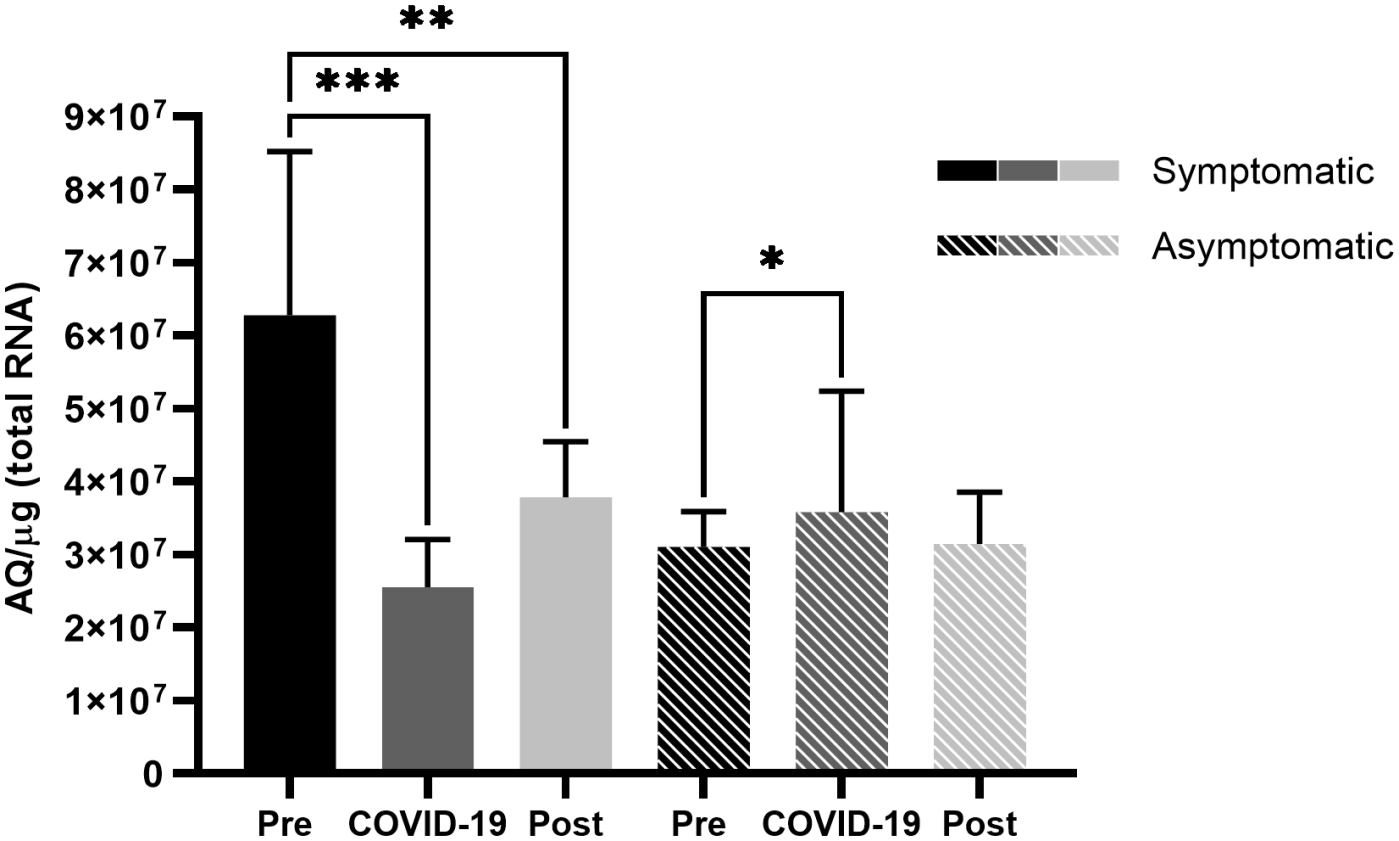
Transmembrane serine protease 2 (TMPRSS2) expression in the nasopharyngeal samples of symptomatic and asymptomatic patients pre-, during-, and post- infection with SARS-CoV-2. Comparison of TMPRSS2 expression levels in symptomatic and asymptomatic patients within different sampling periods. Bars represent Standard Error of the Mean (SEM), and Y axis plots Absolute Quantity per microgram of total RNA (AQ/µg). With the Fisher-Snedecor F Distribution, significant differences were established within the groups, represented by *p < 0.05, **p < 0.01, and ***p < 0.001.

However, symptomatic patients demonstrated high expression levels of TMPRSS2 prior to SARS-CoV-2 infection, which significantly reduced in the presence of the virus (*p* < 0.001) (**Figure 2**), and in contrast to ACE2 levels, TMPRSS2 levels did not return to the levels observed prior to infection, thus demonstrating a difference between pre- and post-infection levels (*p* < 0.01) (**Figure 2**). Asymptomatic patients did not present significant differences in ACE2 levels (**Figure 1**), but there was a significant difference in TMPRSS2 levels before infection and during COVID-19 (*p* < 0.05) (**Figure 2**).

**Figure 3:**
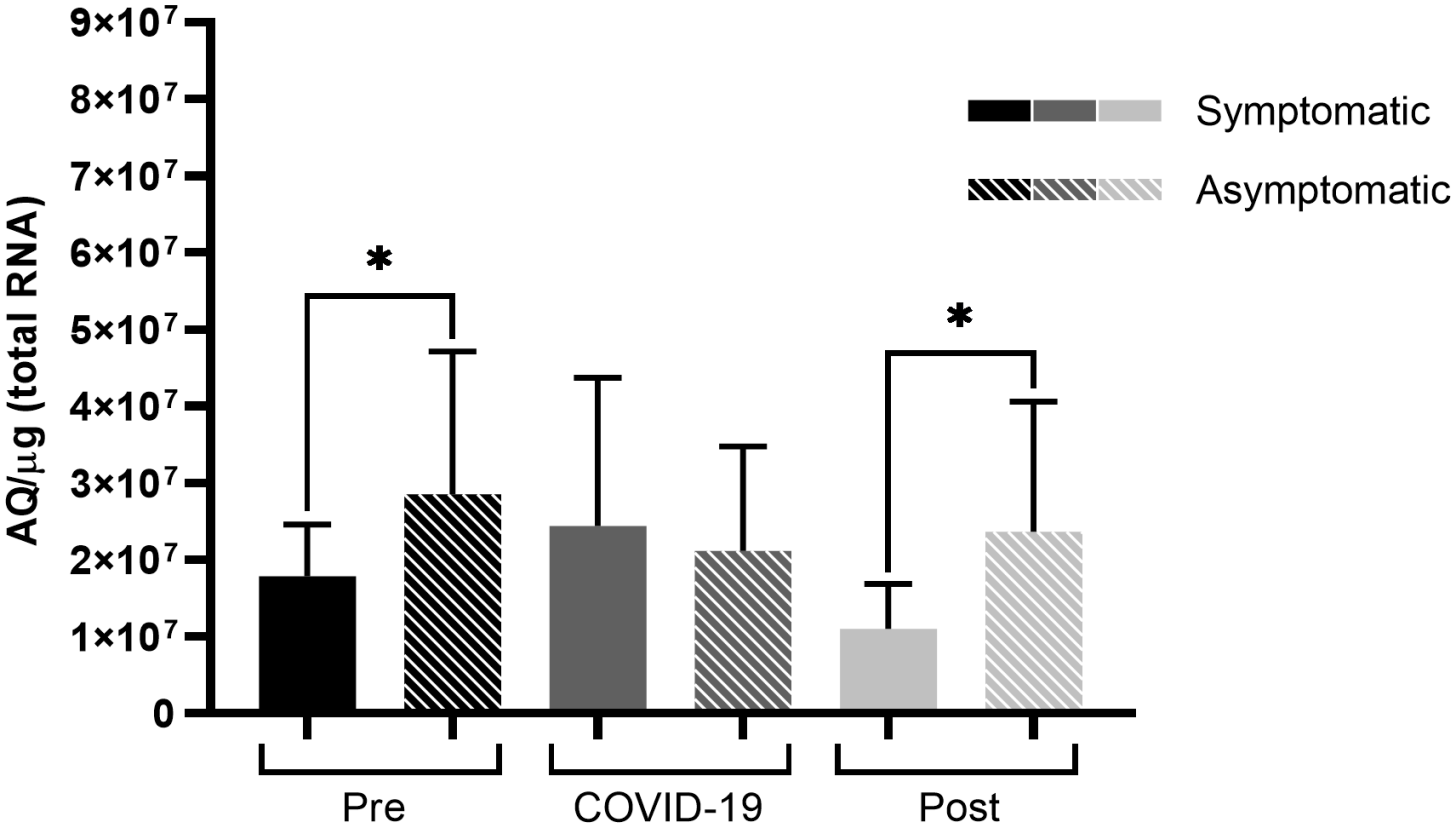
ACE2 expression among symptomatic and asymptomatic patients infected with SARS-CoV-2 at varying sampling points. Comparison of ACE2 expression levels between symptomatic and asymptomatic patients at different sampling points before, during, and after SARS-CoV-2 infection. Bars represent Standard Error of the Mean (SEM), and Y axis plots Absolute Quantity per microgram of total RNA (AQ/µg). Using the Fisher-Snedecor F distribution, significant differences were established within the groups, represented by *p < 0.05.

When comparing symptomatic to asymptomatic patients, those who were symptomatic had significantly lower ACE2 expression levels before infection (*p* < 0.05) than those in asymptomatic patients (**Figure 3**). This difference was maintained during the post-infection period (*p* < 0.05), and no differences were observed during the infection.

**Figure 4:**
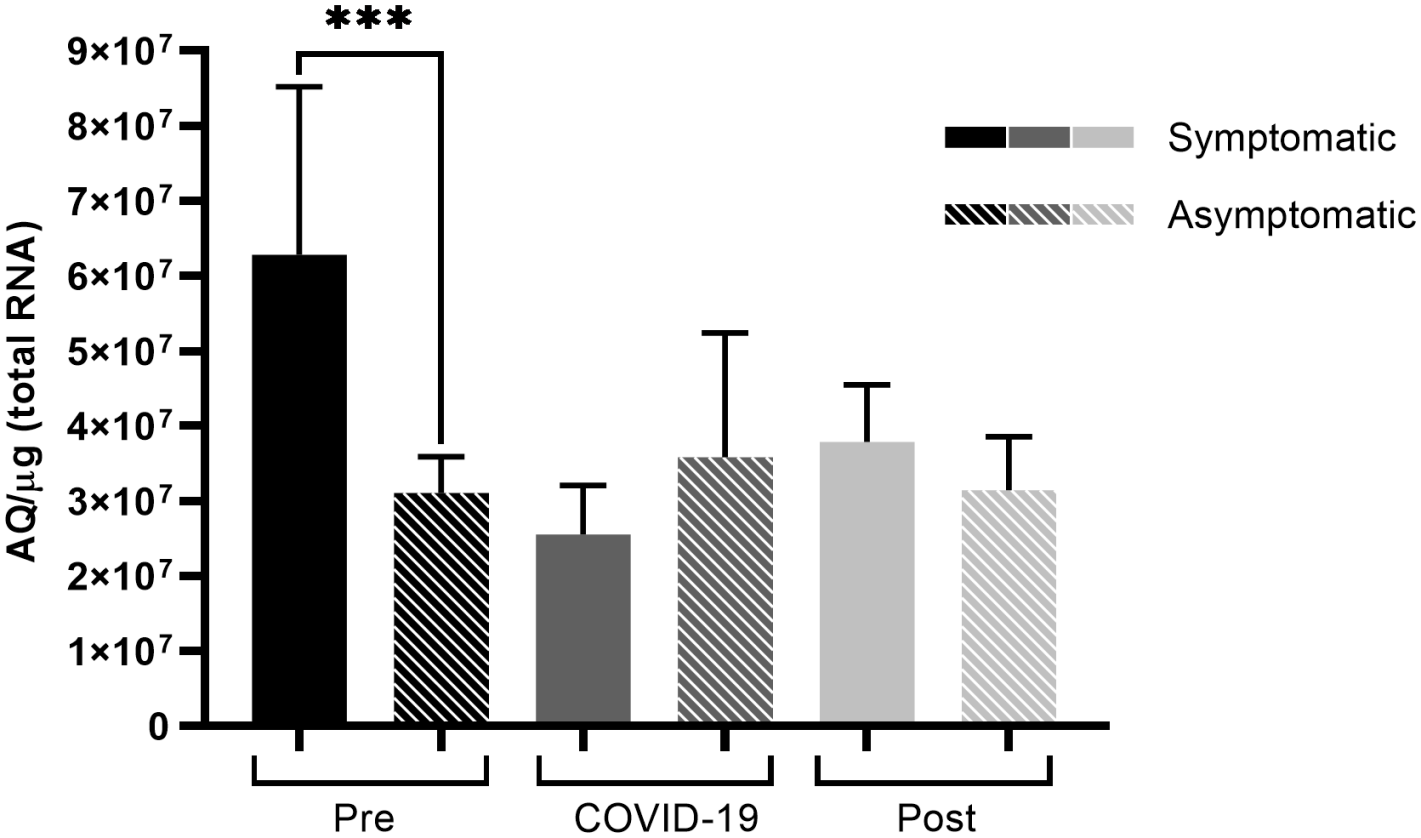
TMPRSS2 expression of symptomatic and asymptomatic SARS-CoV-2 infected patients within the different sampling periods. Comparison of TMPRSS2 expression levels between symptomatic and asymptomatic patients at different sampling periods before, during, and after SARS-CoV-2 infection. Bars represent Standard Error of the Mean (SEM), and Y axis plots Absolute Quantity per microgram of total RNA (AQ/µg). Using the Fisher-Snedecor F distribution, significant differences were established within the groups, as represented by ***p < 0.001.

Comparing TMPRSS2 levels, a significant difference was observed before infection (**Figure 4**), where symptomatic patients had higher protease expression levels (*p* < 0.001) than those among asymptomatic patients. This was the only significant difference observed in TMPRSS2 levels between symptomatic and asymptomatic patients.

## 4 Discussion

### 4.1 *ACE2* expression levels

**Figure 5:**
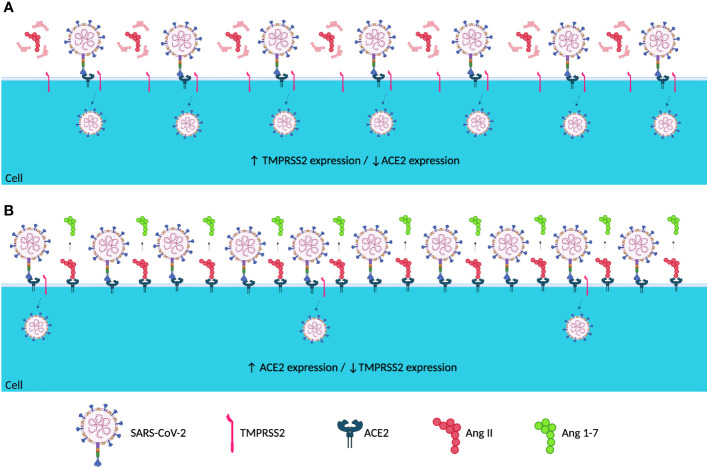
Hypothetical representation of the host cell membrane when infected by SARS-CoV-2 with different patterns of ACE2 and TMPRSS2 expression. Hypothetical representation of the host cell membrane when infected with SARS-CoV-2 in **(A)** low ACE2 and high TMPRSS2 expressions and **(B)** high ACE2 and low TMPRSS2 expressions. In **(A)**, a possible competition between the virus and ang II for the ACE2 receptor occurs, resulting in an increase in viral entry and dissemination. In **(B)**, the hypothetical competition for the ACE2 receptor did not occur, thus decreasing viral dissemination and entry. SARS-CoV-2 is absent during the pre-infection period; however, it was taken into consideration that when the virus entered the host, the host cells had the expression profiles presented in **(A, B)** (image generated in BioRender ^®^). ↓, Decreased Expression; ↑, Increased Expression.

The present study demonstrated that symptomatic and asymptomatic patients presented with different ACE2 and TMPRSS2 expression levels before and after SARS-CoV-2 infection (**Figures 3**, **4**). Asymptomatic patients did not demonstrate any significant differences in levels among the different sampling points; however, symptomatic patients showed lower ACE2 levels before infection, which increased during infection, and lowered post-infection (**Figures 1**, **3**). Ang II, when not converted by ACE2 but by its AT1 receptor, is known to be a potent vasoconstrictor (Deng et al., 2001) and manifests proinflammatory, profibrogenic, and proliferative effects (Turner et al., 2004; Rüster and Wolf, 2006; Benigni et al., 2010; Kuba et al., 2013). With low ACE2 expressions, a possible competition between Ang II and SARS-CoV-2 for the ACE2 receptor may occur. However, the virus has a higher affinity for the receptor, which may result in a lower conversion of ang II into ang-(1-7) (Patel et al., 2015; Sinha et al., 2020; Rodrigues and de Oliveira, 2021), as evidenced in **Figure 5A**.

Ang-(1-7) has effects contrary to those of ang II (Rüster and Wolf, 2006; Kuba et al., 2013); however, low ACE2 expression levels might have reduced the conversion of ang II to ang(1-7). It has been hypothesized that in low ACE2 expression profiles, ang II would accumulate and be converted by its AT1 receptor, which would trigger the previously mentioned proinflammatory, profibrogenic, and proliferative effects (Turner et al., 2004; Rüster and Wolf, 2006; Benigni et al., 2010; Kuba et al., 2013), which concurs with the fact that low ACE2 expression profiles have already been shown as possible contributor to hypertension by triggering an uncontrolled vasoconstriction (Alabasi et al., 2023). **Figure 1** demonstrates that symptomatic patients seem to respond to SARS-CoV-2 by increasing ACE2 expression during infection. This increase could indicate a possible physiological host response to the low conversion of ang II to ang-(1-7), attempting to decrease the harmful effects of ang II conversion by the AT1 receptor (**Figure 5B**).

A notable limitation is that the samples used in this study represented nasopharyngeal cells; however, ACE2 is highly expressed in the human body (Hamming et al., 2007; Hikmet et al., 2020), with abundant expression in the heart and small intestine tissues (Hikmet et al., 2020; Li et al., 2020), as well as the adipose tissue, where the harmful effects of ang II are present in the form of inflammation and adipogenesis (Ting et al., 2023). Because ACE2 is widely expressed in human tissues (Hamming et al., 2004; Ni et al., 2020; Beyerstedt et al., 2021), the potential effects of lower or higher ACE2 expression may occur within other tissues, possibly influencing COVID-19 symptoms, considering that it infects such important tissues to maintain homeostasis (Wu et al., 2020; Gasmi et al., 2021).

### 4.2 *TMPRSS2* expression levels

Similar to ACE2 levels, TMPRSS2 expression levels before infection were different between symptomatic and asymptomatic patients, where symptomatic patients showed high TMPRSS2 expression levels pre-infection (**Figure 4**). Being an essential protein for SARS-CoV-2 cell entry and viral dissemination (Hoffmann et al., 2020; Ni et al., 2020), high TMPRSS2 expression levels before infection observed in this study could indicate easier and faster viral replication and dissemination (**Figure 5A**), which might reflect in the manifestation of symptoms caused by the rapid dissemination of SARS-CoV-2 within human tissues.

Complementing this hypothesis, by analyzing differences in the expression levels of TMPRSS2 in symptomatic patients at different time points (**Figure 2**), a significant reduction was observed in the expression levels during and after infection, possibly demonstrating a host response to fast viral dissemination by reducing the expression of TMPRSS2 (**Figure 5B**). Thus, low TMPRSS2 expression levels may represent a protective factor against the development of COVID-19 symptoms and the infection itself. Moreover, it has already been demonstrated in rats that the pharmacological inhibition of TMPRSS2 is effective against SARS-CoV infection (Zhou et al., 2015).

In asymptomatic patients, an increase in TMPRSS2 levels was observed during infection (**Figure 2**), which could indicate a possible viral modulation of the protease, hence increasing its expression to facilitate viral dissemination. Viral modulation can be conducted by viral nonstructural proteins, which interfere with intracellular signaling (Raj, 2021). The reduction in TMPRSS2 expression post-infection complements this hypothesis by showing that in the absence of SARS-CoV-2, the expression levels are restored to those observed during the pre-infection period. Considering that the samples analyzed in this study were from a period with a higher prevalence of more pathogenic SARS-CoV-2 strains (Padilha et al., 2022) and a lower vaccination rate, the ACE2 and TMPRSS2 levels should be considered together with both these factors.

In the past pandemic years, several studies evaluated the possibility of using ACE2 and TMPRSS2 inhibitors as a treatment for COVID-19. ACE2 inhibition didn´t show promising results, since it could have several side effects given its crucial role in human homeostasis (Donoghue et al., 2000; Santos et al., 2017; Shin et al., 2022), and with the results presented in this manuscript where lower ACE2 levels appeared to be a risk factor for symptomatic COVID-19, inhibiting ACE2 probably wouldn’t be of much help. On the other hand, TMPRSS2 does not have a well-described physiological role within our body (Antalis et al., 2011) and the use of a protease blocker, camostat mesylate, showed encouraging results and it´s still undergoing investigation to be used as an off-label treatment for SARS-CoV-2 infections (Hoffmann et al., 2020; Shapira et al., 2022). Since higher TMPRSS2 levels seemed to be a possible determinant for COVID-19 symptoms in the present study, the usage of protease blockers would show itself to be appealing, although not knowing TMPRSS2´s role in human physiology could be a risk.

The pathogenicity of SARS-CoV-2 is complex and should be taken into consideration, with several factors that could influence and contribute to the successful viral infection and the manifestation of symptoms. Nevertheless, ACE2 and TMPRSS2 have crucial roles in SARS-CoV-2 infection well de-scribed in the literature, and their expression levels appear as a possible factor for the development of COVID-19 symptoms. In this study, it was shown that low expression levels of ACE2 along with high expression levels of TMPRSS2 in nasopharyngeal samples before infection may be a risk factor for the development of COVID-19 symptoms, and SARS-CoV-2 infection seems to increase ACE2 expression and decrease TMPRSS2 levels in symptomatic patients, possibly showing a host response to the replication of SARS-CoV-2. These results can corroborate to the understanding of the viral dynamic of SARS-CoV-2 within the human body, as well as providing an insight on possible COVID-19 symptomatology prediction factors, such as ACE2 and TMPRSS2 expression levels. It is essential that a larger population be evaluated, alongside with RAAS blockers usage, although having longitudinal samples from the same patient in different periods is quite uncommon.

## Data availability statement

The original contributions presented in the study are included in the article/supplementary materials. Further inquiries can be directed to the corresponding author.

## Ethics statement

The studies involving humans were approved by Institutional Ethics Review Board of the Federal University of Santa Catarina (CAAE: 57722022.2.0000.0121). The studies were conducted in accordance with the local legislation and institutional requirements. Written informed consent for participation in this study was provided by the participants’ legal guardians/next of kin.

## Author contributions

HG: Conceptualization, Formal Analysis, Investigation, Methodology, Visualization, Writing – original draft, Writing – review & editing. MS: Conceptualization, Investigation, Methodology, Writing – original draft, Writing – review & editing. FB: Methodology, Writing – original draft, Writing – review & editing. JW: Methodology, Writing – original draft, Writing – review & editing. MV: Methodology, Writing – original draft, Writing – review & editing. GW: Writing – original draft, Writing – review & editing. GF: Conceptualization, Formal Analysis, Methodology, Writing – original draft, Writing – review & editing. MB: Conceptualization, Formal Analysis, Funding acquisition, Investigation, Methodology, Supervision, Visualization, Writing – original draft, Writing – review & editing.
